# Does capitation prepayment based Integrated County Healthcare Consortium affect inpatient distribution and benefits in Anhui Province, China? An interrupted time series analysis

**DOI:** 10.5334/ijic.4193

**Published:** 2019-07-08

**Authors:** Dai Su, Yingchun Chen, Hongxia Gao, Haomiao Li, Liqun Shi, Jingjing Chang, Di Jiang, Xiaomei Hu, Shihan Lei

**Affiliations:** 1 Tongji Medical College, Huazhong University of Science and Technology, CN; 2 Hubei Province Key Research Institute of Humanities and Social Sciences, CN

**Keywords:** capitation prepayment, Integrated County Healthcare Consortium, inpatient distribution and benefits, interrupted time series analysis

## Abstract

**Objective:**

This study aims to compare the level and trend changes of inpatient and funds distribution, as well as inpatient benefits before and after the official operation of the ICHC in Anhui.

**Methods:**

A total of 1,013,815 inpatient cases were collected from the hospitalisation database in two counties in Anhui Province, China, during the course of the study from January 2014 to June 2017. The effect of the reform was assessed beginning with its formal operation in February 2016. Longitudinal time series data were analysed using segmented linear regression of an interrupted time series analysis.

**Results:**

The average hospitalisation expenses showed a decreasing trend and the actual compensation ratio increased significantly (p-value < 0.01). Most of the indicators in the two counties performed well, and the effect of ICHC policy was better in Funan County than in Dingyuan County. The distribution of inpatients and NRCMS funds outside the county after the reform in Dingyuan showed an increasing trend (0.27, 95%CI: 0.12 to 0.42, p-value < 0.01; 0.70, 95%CI: 0.32 to 1.09, p-value < 0.01) and the distribution of inpatients and NRCMS funds in THs showed a more obvious upward trend after the reform in Funan (0.44, 95%CI: 0.22 to 0.67, p-value < 0.001; 0.34, 95%CI: 0.23 to 0.45, p-value < 0.001).

**Conclusions:**

This study suggests that the ICHC policy provides effective strategies in promoting the integration of the healthcare delivery system in China. These strategies include strengthening family doctor signing service system and health management, developing telemedicine technology, reducing the weak points of the healthcare services, and introducing private hospitals to form new ICHCs.

## Introduction

In 1957, the World Health Organization proposed the concept of three-tiered healthcare services (basic, secondary, and tertiary healthcare) and recommended that the concept could be implemented in all countries [1]. In 2009, the Chinese government launched its new nationwide healthcare delivery system reform, including the construction of an integrated healthcare delivery system. At present, a three-tiered healthcare network based on county-level hospitals (CHs), township-level hospitals (THs) and village clinics has been established in rural areas [2]. THs and village clinics mainly provide outpatient services, preventive healthcare, health education, and basic healthcare for some common and frequently occurring diseases. Meanwhile, CHs provide outpatient and inpatient services, basic healthcare for the most common and frequently occurring diseases, and diagnosis and treatment of some perplexing diseases. CHs also provide technical support and personnel training to THs and village clinics. The effective coordination and referral mechanism of the three-tiered healthcare network provides protection for rural residents to obtain appropriate basic healthcare. In 2002, the Chinese government established the New Rural Cooperative Medical System (NRCMS) to provide rural residents with outpatient and inpatient reimbursement for some compliance costs [3] and differentiated hospitalisation compensation levels in different-tiered hospitals to guide the reasonable distribution of inpatients. In 2016, the coverage rate of NRCMS reached 99.36%. The hospitalisation rate of rural residents greatly increased from 3.4% in 2003 to 16.4% in 2016 [4]. After 2016, the NRCMS was gradually merged with the medical insurance system for urban residents in various provinces to form the basic medical insurance system for urban and rural residents.

In the 21st century, international experience has proven that an integrated healthcare delivery system is an effective means to improve the performance of the service delivery system [5]. The payment of medical insurance, a means of effective regulation, plays the roles of ‘leverage’ and ‘engine’ in an integrated healthcare delivery system [6]. On the one hand, as a benefit incentive, the payment directs healthcare providers to collaborate in the best interests of patients. On the other hand, the payment is also easier to operate than other incentives and has, therefore, become a necessary complementary measure in the international integrated healthcare reform. In 2012, the Centers for Medicare and Medicaid Services in the United States began to implement the Accountable Care Organizations (ACOs) [7]. ACOs adopt the group total prepayment system and implements the scheme of ‘hospital funds balance sharing’, which uses low cost to provide healthcare services and management for patients, with the balance of the insurance funds distributed among members in the regional integration service network.

The practice of integrated healthcare delivery system reform began around 2011 in China with urban and rural areas taking the form of a ‘medical commonwealth’ and a vertical collaboration system, respectively [8]. However, in the practice of integrated healthcare delivery system reform in China, the payment of NRCMS is mainly directed at a single hospital or paid-for units, projects, and diseases. As the useful function of controlling cost is established in hospitals, the quality of healthcare is also optimised. However, there has been no orderly guidance for the hospital choices of rural residents and three-tiered healthcare hospitals have yet to achieve a good cooperative relationship with hospitals pursuing the maximisation of their own interests and the healthcare delivery system in a ‘fragmented’ pattern [9]. Given that rural residents in China can freely choose hospitals, the gap in healthcare capabilities of hospitals leads patients to high-level hospitals for improved treatment. This unreasonable distribution reduces the efficiency of healthcare and wastes medical resources, resulting in the rapid increase of healthcare expenses and places tremendous pressure on the NCRMS funds [10]. In 2016, the proportion of patients to primary hospitals decreased by 6.8% in comparison with 2010. This downward trend is more evident in inpatients. In 2016, the proportion of inpatients in primary hospitals decreased by 9.6% in comparison with 2010. The proportion of inpatients in tertiary hospitals increased but decreased in secondary hospitals. The per capita hospitalisation cost of inpatients in tertiary hospitals in 2016 was 12,847.8 yuan, which was more than twice that in secondary hospitals (5,569.9 yuan) [11].

In February 2015, Anhui Province launched the reform of the Integrated County Healthcare Consortium (ICHC) and identified the first batch of 15 pilot counties [12]. The pilot counties then proceeded with program preparation and institutional adjustments and officially operated in February 2016. The specific measures of the reform are as follows: (1) Integrate high-quality healthcare resources in the county, led by CHs and jointly by THs and village clinics and to establish a number of ICHCs that can provide integrated and continuous healthcare for residents within the ICHC. At the same time, form a horizontal competition between different ICHCs. (2) The core of the reform is the capitation prepayment of the NRCMS funds. According to the number of the population covered by the ICHC, the management department of NRCMS pre-packages healthcare costs, including outpatient and inpatient services and referral services, to the lead hospital in ICHC. The NRCMS funds follow the settlement principle of ‘exceeds expenditures does not make up, the balance holds for use’. ICHCs determined the distribution of balance between three-tiered hospitals through performance assessment and realised the integration of the healthcare delivery system and NRCMS payment system.

Although the implementation of ICHC reform in Anhui Province has not been long, some scholars in China have studied the effect of this reform. Wang et al. [13] collected NRCMS data from 2014 to 2016 in Funan County (reformed) and Yingshang County (unreformed) in Anhui and used annual data to compare the trend change and effect of the ICHC before and after the reform in Funan County. A year-by-year decline of the proportion of the expenditure, hospitalization expenses and hospitalization reimbursement by NRCMS outside the county, and an increase of such proportion within the county between 2014 and 2016. Liu et al. [14] analysed the data of NRCMS dataset in Dingyuan County for 2015–2016 through descriptive statistical analysis and found that the number of inpatients seeking treatment outside of the county decreased by 3.31% and the number of inpatients in county- and township- level hospitals were increasing. Yu [15] used medical insurance theory to analyse the changes of medical expenses in ICHC and found that after the reform in Tianchang City in Anhui, the proportion of inpatients in county-level hospitals increases, medical expenses have been further controlled, and the actual reimbursement ratio increases. On the basis of literature research, Zhao [16] used the implementation status and effectiveness of ICHC in Tianchang City of Anhui using ROCCIPI framework, and found that optimizing the distribution mechanism of benefits is the core of reforming sustainable development. Although the ICHC policy has been widely implemented in Anhui, its analysis methods mainly concentrated on theoretical analysis and qualitative research based on descriptive analysis using annual data. Thus, little evidence based on time series data has been published on the effect of this policy. Existing studies on the effect of the evaluation of ICHC show that ICHC reform has positive changes in all indicators [13,14,15,16]. However, we assume that the reform may have certain problems which we verify in this study.

Therefore, this study aims to use the interrupted time series analysis (ITSA) method to compare the changes in the levels and trends of inpatients and NRCMS funds distribution and the benefits of inpatients before and after the implementation of the capitation prepayment based ICHC to identify the strengths and deficits in the reform. We hypothesize that the ICHC reform will significantly improve the distribution of inpatient and fund, and that the benefits will be better. However, due to the defects in the specific ICHC policy design of each county, there may be differences in the effect of the reform, and some indicators may show poor results. The analysis of the reasons for the differences in indicators is the key to futher improve the ICHC policy design.

## Methods and data

### Study design and sampling

In the regional healthcare delivery system reform, the reform of the NRCMS payment system is usually closely related to the medical treatment process (outpatient and inpatient) [17]. Therefore, the rural inpatient data in reform areas were considered as the study object. This study conducted a longitudinal survey on the inpatient data between 1 January 2014 and 30 June 2017 with a quasi-experimental design before and after the policy intervention. The ‘interrupted time point’ was February 2016 when the ICHC reform was formally launched.

**Figure 1:**
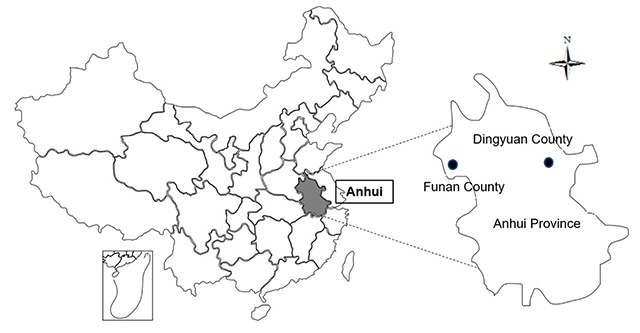
The geolocation of Dingyuan County and Funan County in Anhui Province, China.

Anhui Province is located in Eastern China. We chose Dingyuan County and Funan County, located respectively in the eastern and western part of Anhui province, as our research sample (Figure 1 and Table 1). The two counties were chosen for two reasons. (1) As part of the first batch of pilot counties with capitation prepayment based ICHC reform, the two counties provided a high degree of credibility for assessing the reform effect. (2) Although the capitation prepayment based ICHC reform was performed in the two counties, some differences still exist in specific policies.

**Table 1:** Social economic status and other characteristic of sampling counties in 2016.

	Dingyuan	Funan

Total area (km^2^)	2998.0	1768.4
GDP (billion RMB)	166.3	145.6
Resident population	801000	1183000
Rural population	645732	603345
Per capita disposable income of rural residents (RMB)	10220	10196
Number of CHs	1	4
Number of THs	28	31
Number of ICHCs	1	3
Number of outpatients of NRCMS	101087	203255
Hospital bed utilization ratio (%)	35.64	42.36
Average length of stay (LoS) of rural inpatients (days)	5.77	7.59
Number of health personnel per 1000	5.06	3.30
Number of practicing physician per 1000	1.70	0.83
Number of registered nurse per 1000	1.65	0.75
Number of hospital bed per 1000	3.63	3.14

*Note*: GDP, Gross Domestic Product; RMB, Ren Min Bi; CHs, County-level Hospitals; THs, Township-level Hospitals; ICHCs, Integrated County Healthcare Consortiums; NRCMS, New Rural Cooperative Medical System; LoS, Length of Stay.

### Data and outcome variables

The data were derived from the National Dataset for New Rural Cooperative Medical Insurance in Dingyuan and Funan and were quality-checked by the National Health and Family Planning Commission and hospitals of Dingyuan and Funan. The following information was contained in the hospitalisation database: (1) socio-demographic characteristics, including sex, age, and house address, (2) hospital information, including hospital name and level (hospitals outside the county, CHs and THs), (3) principal diagnosis with the 10th revision of the International Classification of Diseases coding system, and (4) hospitalisation expenses, including total expenses and details, OOP, and actual compensation expenses. Dingyuan and Funan had 343,981 and 669,834 hospitalisation records, respectively, between 1 January 2014 and 30 June 2017. All patient information, such as name, address, and inpatient number was excluded before the data were provided to the study team.

Inpatient distribution refers to the inpatients who have the option to select an hospital, according to their own actual health needs and disease severity to obtain healthcare services. Inpatient benefits refers to the health economic burden of inpatients and the level of actual benefits during the utilisation of healthcare services. In the context of patients having the freedom to choose hospitals and differential compensation system for medical insurance in China, inpatient distribution is often used as an important indicator of the effectiveness of the medical service system reform in China [18]. The resulting change in the distribution of medical insurance funds is related to its efficiency and sustainable development. The change in the level of inpatient benefits is a comprehensive indicator of the integration effect of the medical service system and the health insurance system [19]. Therefore, two kinds of indicators are widely used in China [20,21].

According to the hospitalisation database, the key indicators can be calculated as follows: (1) distribution of inpatients (including the constituent ratio of inpatients outside the county, in CHs and in THs): divide the number of inpatients outside the county/in CHs/in THs by the total number of inpatients, (2) distribution of NRCMS funds (including the constituent ratio of NRCMS funds outside the county/in CHs/in THs: divide the amount of compensation cost outside the county/in CHs/in THs by the total amount of compensation cost of NRCMS funds, and (3) benefits of inpatients (including average hospitalisation expenses and actual compensation ratio): divide each inpatients’ compensation fee of NRCMS funds by their total hospitalisation expenses.

### Statistical analysis

ITSA offers a quasi-experimental research that can effectively evaluate the long-term effects of intervention [22,23]. It comprehensively considers the original trends of things and compares the level and trend before and after the interventions to evaluate the effect of intervention. ITSA is divided into single-group and multiple-group analysis [22]. Multiple-group analysis requires that one or more control groups is available for comparison, whereas single-group analysis does not require a control group under study. ITSA has been proposed as a flexible and rapid design to be considered before defaulting to traditional two-arm, randomly controlled trials. Without a control group, the single-group analysis of ITSA can also obtain robust estimators. Hence, this study used the single-group analysis of ITSA [24,25].

The standard regression model of the single-group ITSA is as follows:

1 {Y_t} = {\beta _0} + {\beta _1}{T_t} + {\beta _2}{X_t} + {\beta _3}{X_t}{T_t} + {\varepsilon _t}

In the above equation, *Y_t_* is the mean number of outcome variables measured, which is an evaluation index that describes study objects in month *t, T_t_* is a time series variable indicating the time in months at time t from the start of the observation period (coding 1, 2, 3, 4 until the last month), *X_t_* is a dummy variable indicating time *t*, which represents ‘0’ if time t occurs before policy intervention and ‘1’ if time t occurs after policy intervention, *X_t_T_t_* is an interaction term calculated by (T –25)×X so that it runs sequentially starting at 1 in this study, and *ε_t_* is the random error term at time t, which represents the part that cannot be explained in the model. In this single-group model, *β*_0_ estimates the intercept or the baseline level of the outcome variable per month at the start time of the observation period, *β*_1_ estimates the slope or trend of the outcome variable before the policy intervention, *β*_2_ estimates the level change of the outcome variable immediately following the introduction of policy intervention, and *β*_3_ estimates the slope or trend change of the outcome variable between pre-intervention and post-intervention. The two parameters (*β*_1_ + *β*_3_) play an important role in estimating the post-intervention slopes or trend.

We used a monthly mean value of outcome variables and determined February 2016 as the intervention time point for the start of capitation prepayment in the integrated healthcare consortium. Segmented linear regression divides the time series into pre- and post-February 2016 segments. Seasonality adjustment was not needed because the autocorrelation function of the outcome variables from 1 January 2014 to 30 June 2017 was tested. To allow for autocorrelation in the data, the official Stata packages ‘newey’ and ‘prais’ were used to fit generalised least-squares (GLS) regression [24]. Two packages allow for fitting a segmented linear regression model under the condition of autocorrelation and controlling for confounding omitted variables. We obtained the order of autocorrelation by examining both the autocorrelation function and the partial autocorrelation function [26]. We used the Durbin–Watson (DW) statistic to test whether the random error terms follow a first-order autoregressive [AR (1)] process [27]. The DW value was 1.3428 and autocorrelated disturbances existed. To confirm whether the outcome variable exists in other ‘interrupted time points’ before or after the true time of intervention, we used the median time point before the true time of intervention as the pseudo-start point to maximise power for testing a significant jump. Stata 15.0 software (Stata Corp LP, College Station, TX, USA) was used for statistical analysis in a Windows environment. The two-sided statistical significance level was set at 0.05.

### Ethical considerations

Approval for this study was obtained from the Ethics Committee of the Tongji Medical College, Huazhong University of Science and Technology (IORG No: IORG0003571).

## Results

### Sociodemographic characteristics of study population

Table 2 displays the details of inpatient characteristics by county, gender, and age before and after the formal operation of the reform in two counties. A total of 1,013,815 cases were collected from the hospitalisation database in two counties. Table 2 shows similar characteristics for both pre-intervention and post-intervention groups compared with those of the total number of inpatient cases.

**Table 2:** Descriptive statistics of inpatient characteristics in two counties.

		ICHC reform*	

Items	Total	before	after

County			
Dingyuan	343,981(33.93)	192,288(34.37)	151,693(33.39)
Funan	669,834(66.07)	367,224(65.63)	302,610(66.61)
Gender			
Male	560,008(55.24)	310,660(55.52)	249,347(54.89)
Female	453,807(44.76)	248,852(44.48)	204,956(45.11)
Age, years			
Less than 20	126,067(12.43)	71,734(12.82)	54,333(11.96)
20–39	186,658(18.41)	111,218(19.87)	75,439(16.61)
40–59	275,778(27.20)	146,158(26.12)	129,620(28.53)
60–79	372,192(36.71)	202,548(36.20)	169,644(37.34)
More than 79	53,120(5.24)	27,853(4.98)	25,267(5.56)
Mean (SD)	48.99	49.92	47.84

*Note*: *We selected February 2016 (formal operation of the reform) as the time point for comparing characteristcs of inpatients before and after the intervention in two counties.

### Overall level of indicators before and after policy intervention

Table 3 shows the details on the overall level of indicators of the pre-intervention and post-intervention groups. In terms of the distribution of inpatients, compared with the overall level before the intervention, the constituent ratio of inpatients outside the county in both counties decreased (–15.89% and –22.36%), respectively. The constituent ratio of inpatients in CHs increased (6.16% and 3.89%), respectively. The constituent ratio of inpatients in THs in Dingyuan decreased (–9.11%). However, Funan showed an upward trend in THs of (18.43%). A similar difference existed between the distribution of inpatients and NRCMS funds. The constituent ratio of NRCMS funds outside both counties declined. The decline rate of the post-intervention group in Dingyuan was significant (–21.54%). The constituent ratio of NRCMS funds in CHs in both counties increased (10.20% and 10.92%), respectively. The constituent ratio of NRCMS funds in THs in the two counties was opposite; Dingyuan decreased (–15.12%), but Funan increased (8.29%). In terms of the benefits of inpatients, the average hospitalisation expenses in Dingyuan increased after the policy intervention (1.52%), but it decreased in Funan (–3.73%), and the actual compensation ratios in both counties increased (15.33% and 9.49%).

**Table 3:** Indicators of overall study population and subgroups of pre-intervention and post-intervention.

Indicators	Regions	Overall study	Pre-intervention	Post-intervention

Constituent ratio of inpatients outside the county (%)	Dingyuan	11.30	12.08	10.16
	Funan	24.20	26.61	20.66
Constituent ratio of inpatients in CHs (%)	Dingyuan	67.67	66.02	70.09
	Funan	52.94	52.12	54.15
Constituent ratio of inpatients in THs (%)	Dingyuan	20.94	21.74	19.76
	Funan	22.86	21.27	25.19
Constituent ratio of NRCMS funds outside the county (%)	Dingyuan	23.22	25.44	19.96
	Funan	38.49	41.00	34.80
Constituent ratio of NRCMS funds in CHs (%)	Dingyuan	69.32	66.57	73.36
	Funan	52.03	49.83	55.27
Constituent ratio of NRCMS funds in THs (%)	Dingyuan	7.39	7.87	6.68
	Funan	9.48	9.17	9.93
Average hospitalisation expenses (RMB)	Dingyuan	5825.21	5789.53	5877.68
	Funan	5195.81	5275.38	5078.79
Actual compensation ratio (%)	Dingyuan	49.31	46.43	53.55
	Funan	54.03	52.03	56.97

### ITSA on the distribution of inpatients in two counties

**Figure 2:**
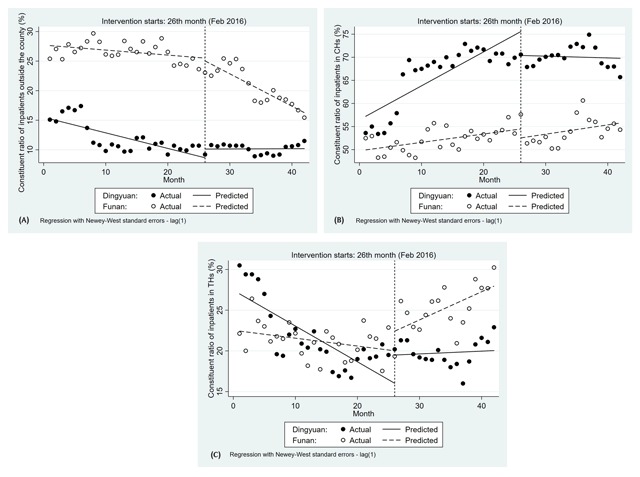
The distribution of inpatients over time. Note: **(A)** Constituent ratio of inpatients outside the county in Dingyuan and Funan (%); **(B)** Constituent ratio of inpatients in CHs in Dingyuan and Funan (%); **(C)** Constituent ratio of inpatients in THs in Dingyuan and Funan (%).

The constituent ratio of inpatients outside Dingyuan County showed a slightly decreasing trend during the period before the policy intervention (–0.26, 95%CI: –0.38 to –0.15, p–value < 0.001). After the policy intervention had been implemented, the constituent ratio of inpatients outside the county changed significantly and showed a slightly increasing trend (0.27, 95%CI: 0.12 to 0.42, p–value < 0.01). Similarly, the constituent ratio of inpatients outside Funan County decreased slightly (–0.08, 95%CI: –0.19 to 0.02, p–value = 0.100). After implementation, the downward trend remained unchanged (–0.46, 95%CI: –0.68 to –0.25, p–value < 0.001) (Figure 2(a) and Table 4).

**Table 4:** Estimated level and trend changes of indicators before and after the intervention in two counties.

	Coefficient (95%CI)			

	Intercept	Preintervention trend	Level change	Trend change

Constituent ratio of inpatients outside the county (%)				
Dingyuan	15.24*** (13.17 to 17.31)	–0.26*** (–0.38 to –0.15)	1.47 (–0.13 to 3.08)	0.27** (0.12 to 0.42)
Funan	27.63*** (26.26 to 28.99)	–0.08 (–0.19 to 0.02)	–0.48 (–3.09 to 2.14)	–0.46*** (–0.68 to –0.25)
Constituent ratio of inpatients in CHs (%)				
Dingyuan	57.21*** (52.37 to 62.05)	0.73*** (0.42 to 1.05)	–5.17* (–9.82 to 0.52)	–0.77* (–1.21 to –0.34)
Funan	49.94*** (47.97 to 51.90)	0.18** (0.06 to 0.30)	–1.93 (–5.17 to 1.31)	0.02 (–0.26 to 0.30)
Constituent ratio of inpatients in THs (%)				
Dingyuan	27.01*** (23.79 to 30.23)	–0.44*** (–0.66 to –0.22)	3.47* (0.13 to 6.81)	0.47** (0.16 to 0.79)
Funan	22.43*** (20.85 to 24.02)	–0.10 (–0.21 to 0.02)	2.41 (–0.46 to 5.28)	0.44*** (0.22 to 0.67)
Constituent ratio of NRCMS funds outside the county (%)				
Dingyuan	31.85*** (26.40 to 37.31)	–0.53** (–0.86 to –0.21)	0.10 (–4.20 to4.40)	0.70** (0.32 to 1.09)
Funan	44.13*** (41.29 to 46.96)	–0.32** (–0.53 to –0.11)	3.56 (–1.58 to 8.72)	–0.30* (–0.71 to 0.11)
Constituent ratio of NRCMS funds in CHs (%)				
Dingyuan	58.40*** (51.43 to 65.37)	0.68** (0.25 to 1.11)	–0.51 (–6.17 to 5.15)	–0.87* (–1.41 to 0.34)
Funan	45.71*** (42.71 to 48.72)	0.40** (0.18 to 0.61)	–3.20 (–8.16 to 1.75)	–0.04* (–0.46 to 0.37)
Constituent ratio of NRCMS funds in THs (%)				
Dingyuan	9.37*** (7.66 to 11.08)	–0.13* (–0.24 to –0.01)	0.25 (–1.57 to 2.06)	0.17* (–0.03 to 0.33)
Funan	10.16*** (9.52 to 10.80)	–0.08*** (–0.12 to –0.04)	–0.37 (–1.32 to 0.59)	0.34*** (0.23 to 0.45)
Average hospitalisation expenses (RMB)				
Dingyuan	5005.05*** (4605.84 to 5404.27)	65.37*** (38.69 to 92.06)	–304.55 (–873.59 to 264.50)	–122.52*** (–186.52 to –58.51)
Funan	5003.84*** (4758.65 to 5249.02)	22.63* (2.81 to 42.45)	–124.69 (–541.19 to 291.81)	–68.39** (–108.35 to –28.43)
Actual compensation ratio (%)				
Dingyuan	52.02*** (49.13 to 54.92)	–0.47** (–0.73 to –0.20)	7.69** (3.33 to 12.05)	1.15*** (0.76 to 1.54)
Funan	51.88*** (50.40 to 53.35)	0.01 (–0.11 to 0.14)	–1.71** (–4.81 to 1.39)	0.80*** (0.57 to 1.03)

*Note*: ***, **, * means statistically significant at the 0.1%, 1% and 5% levels respectively; CI: Confidence interval.

Before February 2016, a consistent upward trend was apparent in the constituent ratio of inpatients in CHs in Dingyuan and Funan (0.73, 95%CI: 0.42 to 1.05, p–value < 0.001; 0.18, 95%CI: 0.06 to 0.30, p–value < 0.01). This policy intervention was associated with a significant decrease in the level (–5.17, 95%CI: –9.82 to 0.52, p–value < 0.05) and slope (–0.77, 95%CI: –1.21 to –0.34, p–value < 0.05) in Dingyuan. However, the constituent ratio of inpatients in CHs in Funan did not change significantly for either level (–1.93, 95%CI: –5.17 to 1.31, p–value = 0.235) or trend (0.02, –0.26 to 0.30, p–value = 0.885) (Figure 2(b) and Table 4).

The segmented linear regression analysis results indicate that prior to the policy intervention in February 2016, the trend of the constituent ratio of inpatients in CHs in Dingyuan significantly declined (–0.44, 95%CI: –0.66 to –0.22, p–value < 0.001). In February 2016, there was an immediate increase in the level of inpatients (3.47, 95%CI: 0.13 to 6.81, p–value < 0.05). The slope of the constituent ratio of inpatients in CHs significantly increased in Dingyuan (0.47, 95%CI: 0.16 to 0.79, p–value < 0.01) and Funan (0.44, 95%CI: 0.22 to 0.67, p–value < 0.001) (Figure 2(c) and Table 4).

### ITSA on the distribution of NRCMS funds in two counties

**Figure 3:**
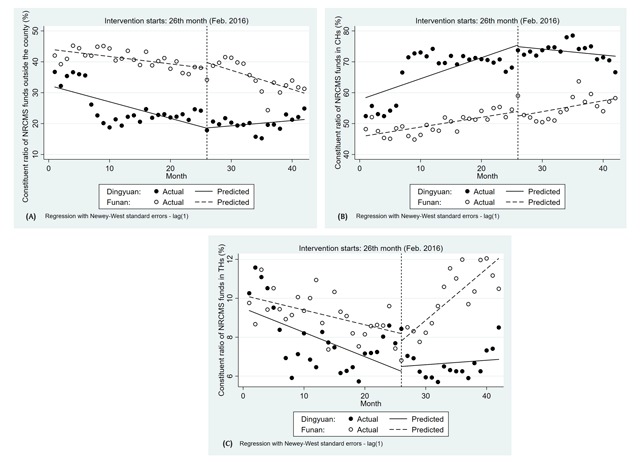
The distribution of NRCMS funds over time. Note: **(A)** Constituent ratio of NRCMS funds outside the county in Dingyuan and Funan (%); **(B)** Constituent ratio of NRCMS funds in CHs in Dingyuan and Funan (%); **(C)** Constituent ratio of NRCMS funds in THs in Dingyuan and Funan (%).

As shown in Figure 3(a) and Table 4, similar to the trend of the constituent ratio of inpatients outside the two counties, the constituent ratio of NRCMS funds outside the county showed a downward trend in Dingyuan (–0.53, 95%CI: –0.86 to –0.21, p–value < 0.01) and Funan (–0.32, 95%CI: –0.53 to –0.11, p–value < 0.01). After the implementation, the trend of the constituent ratio of NRCMS funds outside the county was continuous in Funan (–0.30, 95%CI: –0.71 to 0.11, p–value < 0.05). However, an opposing trend was noted in Dingyuan (0.70, 95%CI: 0.32 to 1.09, p–value < 0.01).

Before the policy intervention, the constituent ratio of NRCMS funds in CHs in both Dingyuan and Funan retained an increasing trend (0.68, 95%CI: 0.25 to 1.11, p–value < 0.01; 0.40, 95%CI: 0.18 to 0.61, p–value < 0.01). This proportion showed a slight decreasing trend in Dingyuan during the period after policy implementation at a rate of –0.19% per month (–0.87, 95%CI: –1.41 to 0.34, p-value < 0.05), but the trend in Funan was continuous after policy intervention at a rate of 0.36% per month (–0.04, 95%CI: –0.46 to 0.37, p-value < 0.05) (Figure 3(b) and Table 4).

A simultaneous slight decrease was observed in the constituent ratio of NRCMS funds in CHs before policy intervention in both counties (–0.13, 95%CI: –0.24 to –0.01, p-value < 0.05; –0.08, 95%CI: –0.12 to –0.04, p-value < 0.001), though there was no significant change in level. Conversely, there was a significant upward trend in the period of post-intervention in Funan (0.34, 95%CI: 0.23 to 0.45, p-value < 0.001) but the change in trend did not differ significantly in Dingyuan (0.15, 95%CI: –0.03 to 0.33, p-value = 0.10) (Figure 3 (c) and Table 4).

### ITSA on the benefits of inpatients in two counties

**Figure 4:**
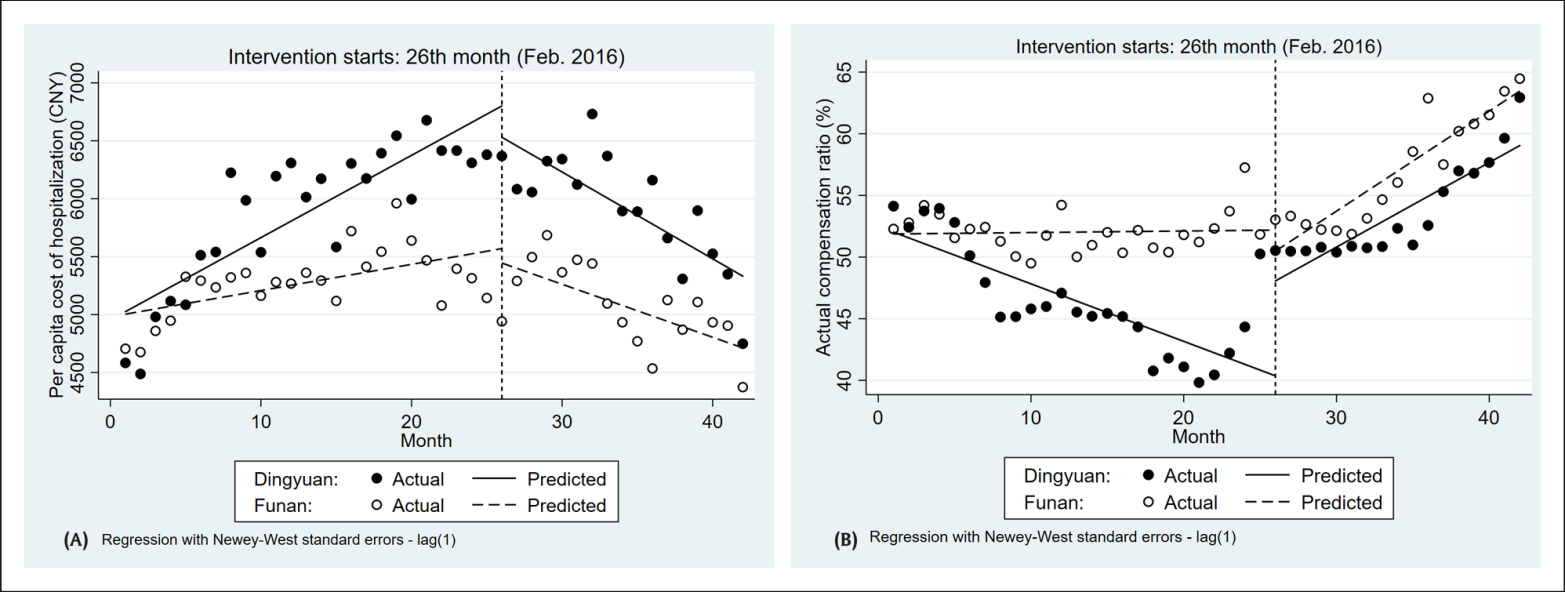
The benefits of inpatients over time. Note: **(A)** Average hospitalisation expenses in Dingyuan and Funan (CNY); **(B)** Actual compensation ratio in Dingyuan and Funan (%).

As presented in Figure 4(a) and Table 4, the segmented linear regression analysis of the average hospitalisation expenses found an upward trend before policy intervention with 65.37 and 22.63 yuan per month in Dingyuan and Funan, respectively (95%CI: 38.69 to 92.06, p-value < 0.001). Although the policy was introduced, the average hospitalisation expenses did not change significantly. However, after the implementation of the policy, the rate remained on a downward trend in the two counties (–122.52, 95%CI: –186.52 to –58.51, p-value < 0.001; –68.39, 95%CI: –108.35 to –28.43, p-value < 0.01).

During the baseline of 25 months before the policy implementation, the actual compensation ratio of inpatients in Dingyuan increased at a rate of 0.47% per month. The intervention was associated with a significant increase in level change (7.69, 95%CI: 3.33 to 12.05, p-value < 0.01) and slope change (1.15, 95%CI: 0.76 to 1.54, p-value < 0.001). Similarly, in Funan, the actual compensation ratio of inpatients also showed an upward trend after policy intervention (0.80, 95%CI: 0.57 to 1.03, p-value < 0.001). However, the level change showed a decrease when the policy was introduced (–1.71, 95%CI: –4.81 to 1.39, p-value < 0.01) (Figure 4(b) and Table 4).

## Discussion

Our research shows that through comparing the trend of changes before and after the formal operation of the capitation prepayment based ICHC in the two counties, significant improvement was observed in the effect of most indicators. A single and ‘fragmented’ system design cannot achieve the purpose of reform. According to a study conducted in Gansu Province [28], the differential hospitalisation compensation ratio between three-tiered hospitals only shows a slight change in patients’ behaviour. Moreover, it is believed that rural residents prefer quality to price when choosing hospitals. This finding is consistent with the empirical evidence provided by PH Brown [29]. The reform of capitation prepayment based ICHC of Anhui shows that the changes in patient behaviour and the improvement of the benefit level are closely related to the integrated design of the system. After the reform of ICHC, the behaviour of patients in the two counties improved markedly, average hospitalisation expenses showed a decreasing trend, and the actual compensation ratio increased significantly. This reform includes the integration and coordination of many policies. The reform of ICHC is based on the integration of the management and payment system. Through the adjustment of the interest distribution mechanism, the common goal incentives and constraints within the ICHC are formed. On the one hand, to maximise the balance of NRCMS funds, healthcare resources (e.g. personnel and equipment) in CHs are utilised by primary care hospitals in the ICHC. Meanwhile, the health demands of rural residents were met through the family doctor signing service system. These measures enhance the capability of THs and reduce the inappropriate medical expenses, as well as promote the first diagnosis of residents in THs, thus increasing the proportion of inpatients in THs and reducing the average hospitalisation expenses. Hence, we suggested that it can develop telemedicine technology [30], share three-tiered resources, and further enhance primary care capability. On the other hand, through payments for disease in the inpatient of capitation prepayment based ICHC and setting the quota of diseases, ICHCs should undertake the excess expenses. CHs and THs are responsible for ‘100 +N’ and ‘50+N’ diseases, respectively (diseases in the two groups are not repeated) and the implementation of a clinical pathway (CP). The reform promoted the hospitals to improve their healthcare capability and is similar to the Kaiser Permanente Model in the United States [31,32,33].

However, the study also found some problems in the ICHC reform, consistent with the research hypothesis. Among these problems is a slight upward trend of the constituent ratio of inpatients and NRCMS funds outside the county (0.27, 95%CI: 0.12 to 0.42, p-value < 0.01; 0.70, 95%CI: 0.32 to 1.09, p-value < 0.01) and a slight downward trend of the constituent ratio of inpatients and NRCMS funds in CHs after the reform (–0.87, 95%CI: –1.41 to 0.34, p-value < 0.05) in Dingyuan. Both results were inconsistent with the reform goals, suggesting that the reform areas were close to large cities with high medical levels and abundant medical resources, which will have a huge ‘syphon effect’ and result in the loss of inpatients in the county. The upward trend of the constituent ratio of inpatients and NRCMS funds in THs is likewise more evident in Funan than in Dingyuan. The result is based on the better policies formulated by Funan in the field of health management. It is suggested that the low ability of health management in Dingyuan will lead patients to seek high-level hospitals. Funan established a leading group for chronic disease management in CHs within ICHCs to carry out health education and health promotion for primary care patients, a health record system and conducted regular follow-ups. Furthermore, Funan carried out the payment of ‘pay for the disease in outpatients’ for chronic and rehabilitation diseases in CHs, thus promoting inpatient to primary care hospitals. This situation is similar to the ACOs, in which the Massachusetts General Hospital conducted a three-year follow-up of elderly chronic disease patients. It was found that for every US $1 that was invested in the Community Resource Commissioner, the cost of medical care saved was US $2.65, though hospitalisation rate was reduced by 20% and the proportion of hospitalised patients with chronic diseases in the community increased significantly [34].

Evidence from the United States, England, the Netherlands, and China indicates that competition among healthcare consortiums can improve health outcomes [35,36,37,38]. Despite the favourable changes of the indicators in this study, positive results were presented at the beginning of the reform. However, the ICHC reform is far from a panacea at present. Take three ICHCs in Funan as an example. If healthcare service capability in different ICHCs cannot match the service population and there is no threshold for patient referral between ICHCs at this stage, when the only motivation for the stronger ICHC is to obtain more patients from the entire region, then the leading ICHC will essentially use this market competition mechanism to expand their strength and profits. Hence, the development of other ICHCs will be inhibited. These alliances are exactly why we should remain cautious about the ICHC policy. Therefore, to form a healthy competition among different ICHCs, patients must be effectively guided to the corresponding ICHC through the family doctor signing service system. The remaining funds of the ICHC should be allocated to strengthen the weak points of the healthcare services and introduce private hospitals to form a new ICHC. The horizontal coordination between ICHCs is reinforced by capitation prepayment and NRCMS compensation design.

## Limitations

This study presents several limitations. First, considering the accessibility of the survey data, we only collected three years of observation from two counties in Anhui Province, where the reform was carried out, and no control group was established in ITSA, and there may be deficiencies in the assessment results. Second, the outcome variables included in this study are mainly related to the distribution of patients and NRCMS funds and the benefit level of patients. However, health outcome indicators, which may be the most important indicator of the effect of reform, are not given sufficient attention. Third, although most of the reform measures are based on capitation prepayment in ICHC, a small number of other measures of reforms also exist, which we did not consider and may lead us to inaccurate results.

## Conclusion

By comparing the results of the two counties, it can be found that the primary health management of inpatients plays an important role in ICHC adjustment and optimization of inpatients and fund distribution, and also has important implications for ICHC reform in other counties. On the one hand, the fund should further encourage the transfer of doctors and medical technology from county-level hospitals to township hospitals, so as to improve the hospitalization service capacity of township hospitals. At the same time, the number of ICHC, the attribute of the lead hospital in ICHC and the suitability of the disease list also need to be paid attention to.
